# The Use of Capsule Endoscopy to Determine Tablet Disintegration In Vivo

**DOI:** 10.3390/pharmaceutics12060498

**Published:** 2020-05-29

**Authors:** Lasse I. Blaabjerg, Li Fan, Xiaoli Chen, Philip J. Sassene

**Affiliations:** 1 Global Research Technologies, Novo Nordisk A/S, Novo Nordisk Park 2, 2760 Maaloev, Denmark; pjsa@novonordisk.com; 2 Novo Nordisk Research Centre China, 20 Life Science Park Road, Beijing 102206, China; lifx@novonordisk.com (L.F.); xach@novonordisk.com (X.C.)

**Keywords:** acetaminophen, pillcam, beagle dogs, tablet disintegration

## Abstract

The preferred delivery route for drugs targeted for systemic effect is by oral administration. Following oral administration, a solid dosage form must disintegrate and the drug dissolve, thereafter permeating the intestinal mucosa. Several different in vitro methods are used to investigate these processes, i.e., disintegration tests, dissolution tests, and permeability models. However, the actual behavior of oral dosage forms in the environment of the gastro-intestinal tract is not very well elucidated using these conventional methods. In this study, the use of capsule endoscopy to determine tablet disintegration in vivo was assessed. Panadol and Panadol Rapid (acetaminophen/paracetamol) were used as the test material. The in vivo tablet disintegration behavior in beagle dogs was assessed by the use of capsule endoscopy. The in vitro tablet disintegration behavior was assessed using the European Pharmacopeia (Ph. Eur.) disintegration test. The study showed that the in vivo disintegration times of Panadol and Panadol Rapid were 24.7 and 16.5 min, respectively, when determined by capsule endoscopy, which corresponded to the pharmacokinetic data. By contrast, the in vitro disintegration times of the same formulations were 5.5 and 4.0 min, respectively, when determined by the Ph. Eur. disintegration test. In conclusion, capsule endoscopy can be used to determine the in vivo tablet disintegration behavior. By contrast, the in vitro methods appear to not be predictive of the disintegration behavior in vivo but may be used to rank the order the formulations with respect to disintegration time.

## 1. Introduction

Oral administration is the preferred delivery route for drugs targeted for systemic effect. Following oral administration, a solid dosage form must disintegrate and the drug dissolve, thereafter permeating the gastric or intestinal mucosa. Several different in vitro methods are used to investigate these processes, i.e., disintegration tests, dissolution tests, and permeability models. The in vitro models vary in complexity from advanced biorelevant models simulating endogenous fluid secretion, motility, and absorption to simple compendial models [1,2,3,4]. However, the actual behavior of an oral dosage form in the environment of the gastro-intestinal tract is not very well elucidated using these conventional methods. Clinical studies can be used to validate the behavior of the dosage form obtained in vitro; however, the characteristics of the gastro-intestinal tract, such as dietary state, gastric emptying rate, motility, and the pH, have all been shown to significantly influence the disintegration, dissolution, and absorption of an orally administered drug [5,6]. 

Focusing only on solid oral dosage forms and the first step toward drug absorption, i.e., the disintegration process, only a few studies have visualized the disintegration in vivo using imaging techniques. X-ray imaging has previously been successfully applied to study the disintegration time and location of differently enteric coated tablets in the gastro-intestinal tract in humans [7]. Another imaging technique, namely γ-scintigraphy, has previously been used to study the disintegration time of Panadol and Panadol Rapid in humans. The study showed that Panadol Rapid disintegrated faster compared to Panadol, which, in turn, was linked to faster uptake of the drug [5]. Similar results were seen in a study using magnetic resonance imaging (MRI) to study the disintegration and transit time of Panadol and Panadol Rapid [8]. Panadol Rapid disintegrated fully in the stomach, whereas Panadol remained intact until complete gastric emptying. Panadol was assumed to have fully disintegrated in the intestine, as the tablet was no longer detectable by MRI and no visual remnants were present in the feces. 

Balloon endoscopy has successfully been used to study the disintegration behavior of a tablet and capsule containing pivmecillinam and pivampicillin, respectively [9,10]. It was shown that the tablet formulation disintegrated more rapidly compared to the capsule formulation. Furthermore, the endoscopy revealed that the volunteers who received the capsule formulation developed gastro-intestinal bleeding and mucosal erosion, whereas this did not occur with the volunteers who received the tablet [10]. Finally, capsule endoscopy has previously been used to determine the rupture time and dispersion of capsules. The study showed that the method could visualize the behavior of a capsule in the human stomach; however, the study did not investigate the influence of the camera capsule on the behavior of the capsule formulation in vivo [11].

As described, several different methods have been used to indirectly or directly visualize the behavior of an oral solid dosage form in the gastro-intestinal tract. Indirect visualization methods include γ-scintigraphy, X-ray imaging, and magnetic resonance imaging (MRI) [5,8,12,13]. X-ray imaging and γ-scintigraphy are simple, well-understood methods to acquire high-resolution images. However, the major drawback of these two methods is the large radiation exposure during repeated imaging, while γ-scintigraphy also requires alteration of the formulation, which may alter drug exposure. MRI is a radiation-free alternative that also provides high-resolution images. However, the drawbacks of this method are, firstly, that the image acquisition time is at least 20 s, during which motion must be avoided to obtain a high resolution of the images; this means real-time imaging is impossible. Secondly, the necessary suppression of gastro-intestinal motility can only be achieved by the use of anticholinergic drugs, which, in turn, alters the environment of the solid dosage form, thereby leading to potentially biased results [8]. Finally, magnetic imaging has also been used to study the transit and disintegration time of magnetic tablets in humans in real time. However, this method also has several drawbacks including the need for the volunteer to be lying down and the need to include magnets in the formulation, thereby potentially altering the behavior of the formulation in the gastro-intestinal tract [14]. Finally, another major drawback for these conventional visualization methods is the limited availability.

Balloon endoscopy is a direct visualization method, which provides images in real time. However, the method can be considered invasive as volunteers may experience significant discomfort during the procedure as the endoscope has to pass through the esophagus into the stomach and, therefore, sedation may be needed [15]. Furthermore, in anaesthetized animals, there is a significant risk of perforation of the gastro-intestinal tract or bleeding due to insertion of the endoscope [16,17]. Additionally, during the procedure, the stomach is inflated with air, which may influence the behavior of a dosage form, and the subject must lie down, leading to potentially biased results [10]. Finally, administration of water, which is recommended upon administration of oral dosage forms, is restricted during the balloon endoscopy [10]. In pharmaceutical development, methods to study the behavior of dosage forms in the gastro-intestinal tract with limited alterations to the formulation and without affecting the physiological process are required. Capsule endoscopy is a minimally invasive method to visualize the gastro-intestinal tract in real time, which requires little-to-no alteration of the studied dosage form. Therefore, this study investigates the use of capsule endoscopy to determine tablet disintegration in beagle dogs by dosing two commercially available tablets with proven differences in the pharmacokinetic profile. The two formulations are dosed to non-sedated beagle dogs. Furthermore, the pharmacokinetic profile of the drug with and without the camera capsule is determined to ensure that the camera capsules do not affect the pharmacokinetic profile of the drug.

## 2. Materials and Methods 

### 2.1. Materials

Two different oral tablet formulations of paracetamol were investigated: Panadol tablets (500 mg paracetamol/tablet; GlaxoSmithKline) and Panadol Rapid tablets (500 mg paracetamol/tablet; GlaxoSmithKline). The excipients of each of these formulations are shown in Table 1. Simulated intestinal fluid (SIF) powder was obtained from Biorelevant (South Croydon, UK). Sodium chloride, sodium dihydrogen phosphate monohydrate, and sodium hydroxide were obtained from Merck Millipore (Darmstadt, Germany).

### 2.2. Determination of Tablet Disintegration Time of Paracetamol Formulations

The disintegration time in water of 6 tablets of Panadol and Panadol Rapid were determined using the method described in the European Pharmacopeia (Ph. Eur.) Section 2.9.1.

### 2.3. Preparation of FaSSIF

Fasted state simulated intestinal fluid (FaSSIF) was prepared from a commercially available SIF powder as specified by the manufacturer (Biorelevant, South Croydon, UK). Phosphate buffer was prepared with 3.954 mg/mL monobasic sodium phosphate, 0.420 mg/mL sodium hydroxide, and 6.186 mg/mL sodium chloride dissolved in purified water (MilliQ Advantage A10, Merck Millipore, Darmstadt, Germany) and adjusted to pH 6.5 using either 1 M sodium hydroxide or 1 M hydrochloric acid. SIF powder was dissolved in the phosphate buffer (2.24 mg/mL) and stirred for 2 h at ambient temperature before use.

### 2.4. Determination of Dissolution Rate of Paracetamol Formulations in FaSSIF

Dissolution of the Panadol and Panadol Rapid was carried out in triplicate at 37.0 ± 0.5 °C using an USP II apparatus (Vision 8 Elite, Hanson Research, Chatsworth, CA, USA) and 500 mL FaSSIF pH 6.5. The paddle speed was set to 50 rpm and a syringe pump (Vision autoplus, Hanson Research, Chatsworth, CA, USA) was used to sample 1 mL (8.5 mL up-take volume) through in-line filtration (Millipore Millex-HV PVDF, 0.45 μm) at time points 5, 10, 15, 20, 30, 45, and 60 min without replacement. Samples were analyzed by HPLC-UV using a Waters Alliance 2690 system containing a quaternary solvent pump, automated sample manager, column oven, and UV detector (Waters 2489 module). The chromatographic separation was conducted on an Aeris Widepore XB-C8 (3.6 µm, 150 × 2.1 mm) column from Phenomenex (Torrance, CA, USA). The mobile phase was acetonitrile:50 mM potassium dihydrogen phosphate monobasic buffer (1:9). The column was maintained at 40 °C and the flow rate and injection volume were 0.4 mL/min and 3 μL, respectively. The UV detection was carried out at 215 nm and quantification was performed using the peak areas of a two-point linear standard curve at 1 mg/mL. The data were processed using Waters Empower 3 software.

### 2.5. Determination of Pharmacokinetic Profiles of Paracetamol Formulations

Two groups of beagle dogs, three for each, with a mean (±s.d.) age of 4.1 ± 0.7 y and a mean weight of 12.3 ± 0.9 kg, were fasted for at least 18 h and restricted access to water 1 h before study started. Each group of dogs were dosed with either Panadol or Panadol Rapid. The drug was orally administered with 50 ml water and the dogs were awake for the entire duration of the study. Venous blood samples were taken at 3, 5, 7.5, 10, 15, 20, 25, 30, 45, 60, 90, 120, 150, 180, and 240 min after drug administration. The plasma samples were analyzed by the fully automated Cobas C501 using reagent cat# 20767174 322 as specified by the manufacturer (Roche Diagnostics, Mannheim, Germany). The quantification was performed using a 2-point linear standard and the measuring range was 1.2–500 μg/mL as given by the manufacturer. The pharmacokinetic parameters were calculated by the use of the software Phoenix 64 version 6.4.0.7968. The dog PK study protocol was approved by the IACUC at Novo Nordisk Research Centre China and the Ethical Review Committee in Novo Nordisk A/S, Denmark (Project code: 2018-09-LIFX; Date: 26th April 2018). The pharmacokinetic profile was obtained in the same dog study as the in vivo tablet disintegration was visualized. The influence of the capsule endoscopy on the pharmacokinetic profile of a paracetamol formulation was determined by dosing the formulation without the camera capsule in the same three dogs.

### 2.6. Determination of Tablet Disintegration Time in Beagle Dogs

The capsule endoscopic visualization studies were carried out in two groups of three beagle dogs each with a mean (±s.d.) age of 4.1 ± 0.7 y and a mean weight of 12.3 ± 0.9 kg, which were fasted for at least 18 h and restricted access to water 1 h before the study started.

**Figure 1:**
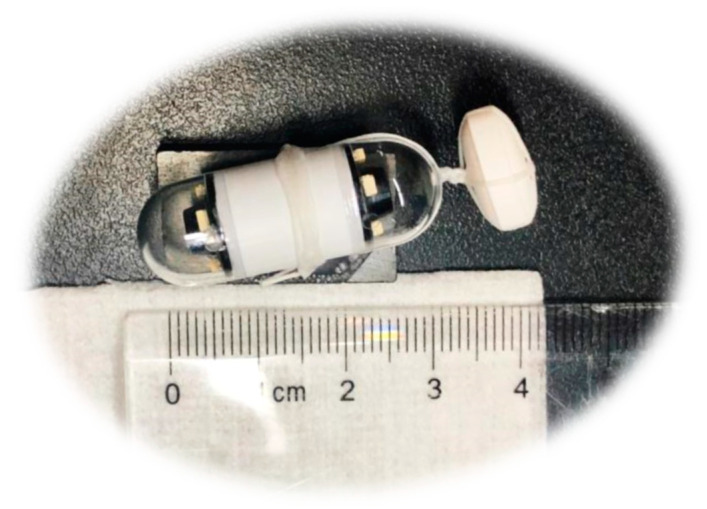
A dosage form is fixed in front of the camera capsule with a soft string (dental floss).

An electrode belt was fastened around the stomach of the respective dogs. The drug formulation was tightened to the camera capsule using a soft string of dental floss (Colgate Total) (Figure 1). The tablet disintegration time was measured from when the tablet entered the dog stomach to when the tablet could no longer be visually observed and was discarded. The dogs were single-housed following dosing to ensure excretion of the camera capsule. All dogs excreted the capsule intact within 24 h after dosing as the camera capsule travelled with the peristaltic movement through the gastro-intestinal tract.

### 2.7. Statistical Analysis

Analysis of variance (ANOVA) was performed using Microsoft Excel 2013 (Microsoft, Redmond, WA, USA). A statistical p value < 0.05 was considered significant. Results are given as mean ± standard deviation, unless otherwise stated.

## 3. Results

### 3.1. Determination of Disintegration Time and Dissolution Rate of Paracetamol Formulations

**Figure 2:**
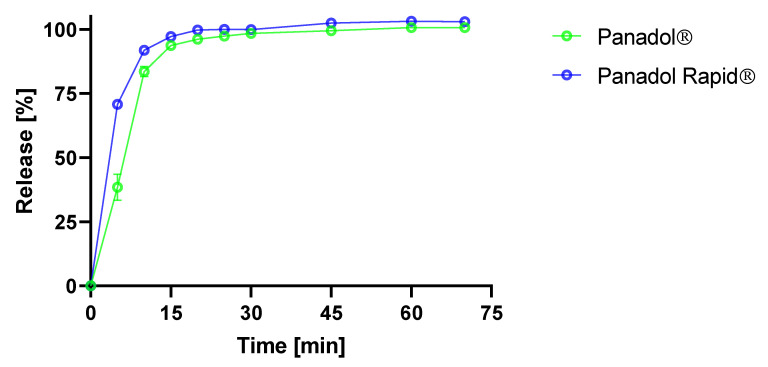
Time–concentration profiles of Panadol and Panadol Rapid when analyzed by the USP II dissolution test (n = 3).

In this study, the in vitro disintegration times of Panadol and Panadol Rapid were 5.5 ± 0.5 and 4.0 ± 1.0 min (n = 6), respectively, when determined by the Ph. Eur. disintegration test. ANOVA was performed on the disintegration times of the two formulations and showed a statistically significant faster disintegration for Panadol Rapid compared to Panadol (p < 0.0001). This can be explained by Panadol Rapid containing sodium bicarbonate, which produces a porous tablet structure when immersed into an aqueous medium. Furthermore, the in vitro time–concentration profiles of Panadol and Panadol Rapid were determined using the Ph. Eur. USP II dissolution test. The results showed that Panadol Rapid released 70.7% ± 2.6% of paracetamol at 5 min, whereas Panadol released 38.5% ± 5.1% of the dose at the same time point (Figure 2). ANOVA was performed on the time–concentration data and showed a statistically significant faster release for Panadol Rapid compared to Panadol (p < 0.005), which can be explained by the faster tablet disintegration of Panadol Rapid. 

### 3.2. Determination of Tablet Disintegration Time in Beagle Dogs

The camera capsule was originally developed for endoscopy to study diseases in the gastro-intestinal tract such as Crohn’s disease and colitis [18,19]. 

**Figure 3:**
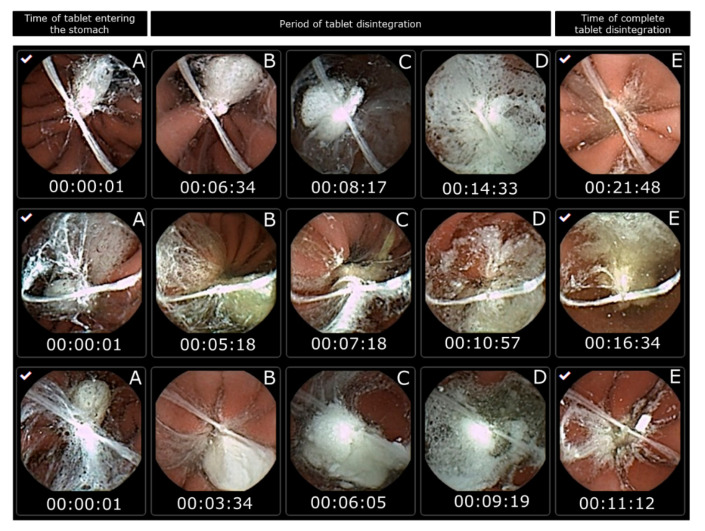
Images from capsule endoscopy to visualize Panadol Rapid disintegration time in each beagle dog after oral administration (n = 3). The time of the tablet entering the stomach is set as the start time (**A**). The period of tablet disintegration is while the tablet is still visible (**B**–**D**). The time of complete tablet disintegration is when no residue is seen of the tablet or the tablet is fully fragmented (**E**).

**Figure 4:**
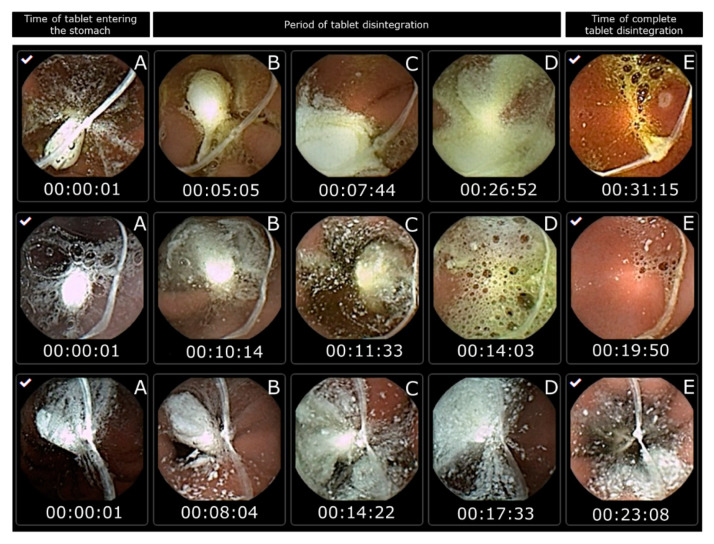
Images from capsule endoscopy to visualize Panadol disintegration time in each beagle dog after oral administration (n = 3). The time of the tablet entering the stomach is set as the start time (**A**). The period of tablet disintegration is while the tablet is still visible (**B**–**D**). The time of complete tablet disintegration is when no residue is seen of the tablet or the tablet is fully fragmented (**E**).

However, in this study, capsule endoscopy was used to study the tablet disintegration of Panadol and Panadol Rapid in beagle dogs. It can be observed that all the tested tablets are submerged in gastric fluid for their entire residence in the stomach. When the tablets disintegrated, their content spread over a large surface area of the epithelial lining covering the stomach. The tablet disintegration time was determined as the time from the tablet entering the stomach to when it was no longer visible from the camera capsule or when the tablet became fully fragmented. The average disintegration time for Panadol and Panadol Rapid was 24.7 ± 4.8 and 16.5 ± 4.3 min, respectively, as can be seen from Figure 3 and Figure 4. 

### 3.3. Determination of PK Profiles of Paracetamol Formulations

As shown in Figure 2, the in vitro dissolution study of Panadol Rapid showed a faster release of the paracetamol compared to Panadol. The two tablet formulations were orally administered in three beagle dogs each.

**Figure 5:**
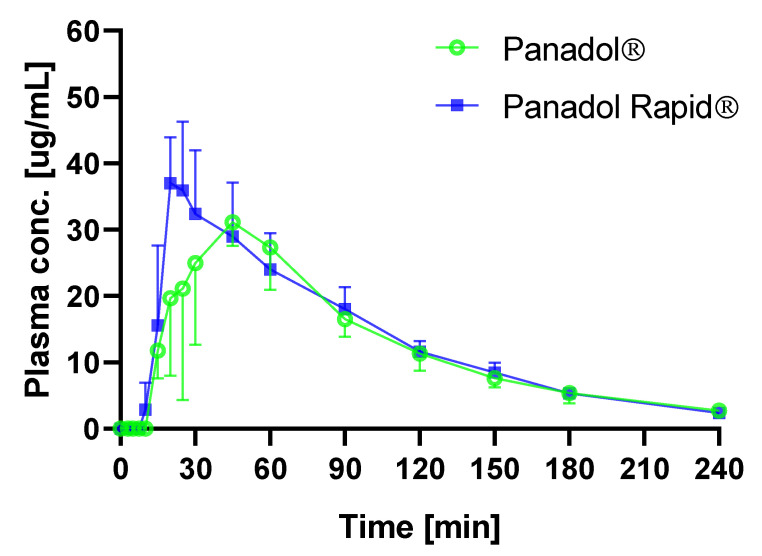
Plasma concentration of paracetamol in beagle dogs after oral administration of Panadol (o) and Panadol rapid (■) (mean ± standard deviation, n = 3).

The average time–plasma concentration profiles when dosing Panadol and Panadol Rapid with capsule endoscopy are shown in Figure 5. Pharmacokinetic data are given in Table 2. When Panadol and Panadol Rapid were administered with capsule endoscopy, the T_max_ were 0.72 ± 0.33 and 0.36 ± 0.06 h, respectively, and the AUC/dose were 1.28 ± 0.09 and 1.38 ± 0.25 h·kg/L, respectively.

ANOVA was performed on the dose-corrected time–plasma concentration data and showed no statistically significant difference between AUC/dose of the two formulations (p = 0.683), which means they gave the same exposure. 

### 3.4. Determination of PK Profiles with and without Capsule Endoscopy

No previous studies in the literature have investigated the effect of capsule endoscopy on the pharmacokinetic profile of a drug. However, the rupture time of a capsule using capsule endoscopy has previously been studied in humans. In that study, an in vitro dissolution study was used to conclude that the capsule endoscopy did not affect the rupture time of the capsule [11].

**Figure 6:**
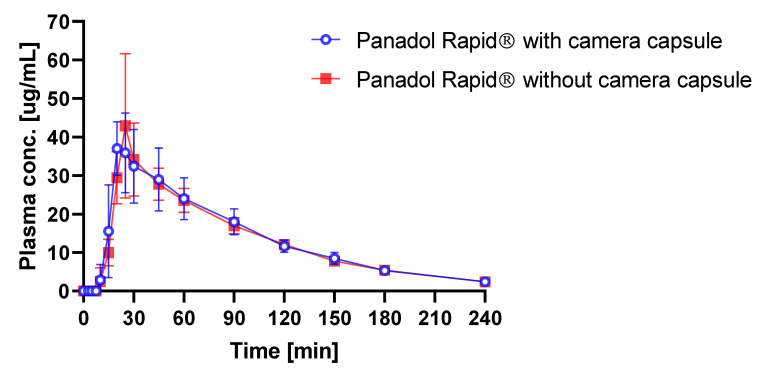
Plasma concentration of paracetamol in beagle dogs after oral administration of Panadol Rapid with (o) and without (■) capsule endoscopy (mean ± standard deviation, n = 3).

It is, therefore, of interest to study the influence of capsule endoscopy on the plasma concentration profile following oral dosing in beagle dogs. The average time–plasma concentration profiles when dosing Panadol Rapid with and without capsule endoscopy are shown in Figure 6. Pharmacokinetic data are given in Table 2. When Panadol Rapid was administered with and without capsule endoscopy, the C_max_ were 40.7 ± 12.20 and 40.2 ± 22.60 mg/L, respectively, the T_max_ were 0.36 ± 0.06 and 0.38 ± 0.06 h, respectively, and the AUC/dose were 1.38 ± 0.25 and 1.12 ± 0.18 h·kg/L, respectively.

ANOVA was performed on the dose-corrected time–plasma concentration data and showed no statistically significant difference in the C_max_, T_max_, and AUC/dose (p = 0.80, p = 0.51, and p = 0.61, respectively) when administering Panadol Rapid with and without capsule endoscopy.

## 4. Discussion

The disintegration behavior of oral dosage forms in the environment of the gastro-intestinal tract is not very well elucidated using conventional disintegration tests. The Ph. Eur. tablet disintegration test is mainly used for quality control purposes and may not adequately simulate the in vivo environment with respect to the differences in available volume and motility. The volume of media in the Ph. Eur. disintegration test is significantly larger, and the motility is more vigorous, which can result in a faster tablet disintegration time and, therefore, potentially faster drug release compared to in vivo [20]. 

The in vitro disintegration times of Panadol and Panadol Rapid were 5.5 ± 0.5 and 4.0 ± 1.0 min, respectively, when determined by the Ph. Eur. disintegration test. By contrast, the in vivo disintegration times for the same formulations were 24.7 ± 4.8 and 16.5 ± 4.3 min, respectively, when determined by capsule endoscopy in beagle dogs. As hypothesized, the in vivo tablet disintegration time was longer compared to tablet disintegration in vitro. Furthermore, it was shown that the Panadol Rapid formulation disintegrates faster compared to the Panadol formulation, which resulted in a shorter T_max_. The faster disintegration time of Panadol Rapid compared to Panadol has also been seen in humans using MRI and γ-scintigraphy [5,8]. The capsule endoscopy showed that the tablets were submerged in the gastric fluid for their entire residence in the stomach. Hence, the method gives an impression of both the tablet disintegration time and local environment of the tablet in the gastro-intestinal tract, such as the amount of fluid and position, which is not possible to the same degree using either MRI or γ-scintigraphy.

To investigate the influence of the capsule endoscopy on the tablet performance in vivo, Panadol Rapid was orally dosed with and without capsule endoscopy in beagle dogs. It was speculated that the use of dental floss to fixate the formulation to the camera capsule and to retain the camera capsule and the formulation in the stomach might reduce the intragastric motility of the investigated formulation. However, ANOVA of the pharmacokinetic parameters C_max_, T_max_, and AUC/dose of Panadol Rapid showed that the drug exposure was not influenced when the tablets were dosed with and without capsule endoscopy (p = 0.80, p = 0.51, and p = 0.61, respectively), which indicates that capsule endoscopy does not affect tablet performance and, thus, tablet disintegration, significantly.

Alternative methods to visualize tablet disintegration, transit, and drug delivery in vivo include magnetic resonance imaging (MRI), γ-scintigraphy, and balloon endoscopy. The methods are considered to be invasive and often require the subject to be anaesthetized [5,8,21]. By contrast, capsule endoscopy presents several advantages including being a non-invasive procedure that can be conducted in unanesthetized animals, giving an increased angle of view, increased number of images, and enables a longer duration of the visualization procedure up to 8 h. This makes the technique ideal for visualizing drug delivery systems in the stomach and in the duodenum [11,22,23]. Furthermore, using capsule endoscopy requires only limited alteration of the formulation by fixating the camera capsule to the solid dosage form, which have been shown not to affect drug exposure in the current study. Although, several studies have shown that a capsule device of the same proportions does not affect the gastric emptying rate and motility [24,25]. It may be speculated that the camera capsule can affect the gastric emptying rate of solid dosage forms of much smaller size with respect to the camera capsule size. For a gastro-resistant coated formulation, prolonged residence time in the stomach could lead to premature release of the drug and severely impact the pharmacokinetics.

It is well known that stress in the animal can induce biased results. In the current study, the sensory belt, which is a belt the ingested camera capsule transmits data to, could be worn by the dogs without the animals feeling discomfort or trying to remove it. Furthermore, the dogs were able to swallow the tablet formulation fixated to the camera capsule and the capsule could transmit data to the sensory belt and receiver. The tablet disintegration could be visualized and quantified in awake animals and no damage from the ingestion of the camera capsules was discovered in the gastro-intestinal tract in the dogs after repeated dosing (data not shown). 

Capsule endoscopy appears to be a promising technology that provides a direct method to visualize and investigate sophisticated drug delivery systems in the gastro-intestinal tract. The method serves as a powerful tool in designing formulations as it offers clear guidance for formulation scientists. An example could be visualization of a gastro-resistant coated tablet in vivo to study coating integrity and tablet disintegration upon its transit from the stomach to the intestine. Additionally, data obtained by capsule endoscopy can be used in development of predictive in vitro models. Moreover, the method can also be used as a control to support the reliability of in vitro studies and to increase the understanding of the conditions that dosage forms are subjected to in vivo. Finally, Russell and Burch described the 3Rs (replacement, reduction, and refine) as guiding principles for more ethical use of animal testing [26]. Capsule endoscopy serves as a refinement and reduction of the animal studies, as the technique allows for coupling of in vivo tablet visualization with pharmacokinetic results and a direct proof to distinguish between different formulations. This can provide a guide for formulation design, which consequently reduces the amount of in vivo studies.

## 5. Conclusions

The use of capsule endoscopy to determine tablet disintegration of Panadol and Panadol Rapid in awake beagle dogs was investigated. It was shown that the in vivo disintegration times of Panadol and Panadol Rapid were 24.7 ± 4.8 and 16.5 ± 4.3 min, respectively. In turn, the faster tablet disintegration for Panadol Rapid resulted in a faster T_max_ of 0.36 h compared to the T_max_ of 0.72 h for Panadol. Furthermore, ANOVA of the pharmacokinetic parameters C_max_, T_max_, and AUC/dose for Panadol Rapid dosed with and without capsule endoscopy (p = 0.80, p = 0.51, and p = 0.61, respectively) indicated that capsule endoscopy does not affect tablet performance and, thus, tablet disintegration. The capsule endoscopy method was well tolerated by the animals, and the tablet disintegration process could be followed visually. In conclusion, this technology provides a direct method to investigate drug delivery systems in the gastro-intestinal tract and may assist the design of new formulations during drug product development.

## Figures and Tables

**Table 1:** Excipients in Panadol and Panadol Rapid.

Formulation	Excipients
Panadol (paracetamol 500 mg)	Maize starch, pregelatinized starch, povidone, potassium sorbate, talc, stearic acid, hypromellose triacetate
Panadol Rapid (paracetamol 500 mg)	Maize starch, pregelatinized maize starch, povidone, potassium sorbate, sodium bicarbonate, microcrystalline cellulose, magnesium stearate, carnauba wax, coating: Titandioxide, polydextrose, hypromellose, triacetate, macrogol

**Table 2:** Summary of pharmacokinetic variables (mean ± 95% confidence interval, n = 3).

Data	Panadol (+Camera Capsule)	Panadol Rapid	Panadol Rapid (+Camera Capsule)
Dose (mg/kg)	40.91 ± 3.49	43.45 ± 8.57	40.50 ± 3.71
T_½_ (h)	0.92 ± 0.33	0.85 ± 0.14	0.89 ± 0.19
T_max_ (h)	0.72 ± 0.33	0.38 ± 0.06	0.36 ± 0.06
C_max_ (mg/L)	36.9 ± 8.49	40.2 ± 22.60	40.7 ± 12.20
C_max/dose_ (kg/L)	0.9 ± 0.23	0.91 ± 0.41	1.01 ± 0.31
AUC_last_ (h·mg/L)	49.05 ± 9.18	46.48 ± 13.40	52.91 ± 14.60
AUC_inf_ (h·mg/L)	52.39 ± 8.06	49.03 ± 14.2	56.02 ± 13.70
AUC/dose (h·kg/L)	1.28 ± 0.09	1.12 ± 0.18	1.38 ± 0.25
